# Metasurface Fabrication by Cryogenic and Bosch Deep Reactive Ion Etching

**DOI:** 10.3390/mi12050501

**Published:** 2021-04-29

**Authors:** Angela M. Baracu, Christopher A. Dirdal, Andrei M. Avram, Adrian Dinescu, Raluca Muller, Geir Uri Jensen, Paul Conrad Vaagen Thrane, Hallvard Angelskår

**Affiliations:** 1 National Institute for Research and Development in Microtechnologies-IMT Bucharest, 126A, Erou Iancu Nicolae Street, 077190 Voluntari, Romania; andrei.avram@imt.ro (A.M.A.); adrian.dinescu@imt.ro (A.D.); raluca.muller@imt.ro (R.M.); 2 SINTEF Microsystems and Nanotechnology, Gaustadalleen 23C, 0737 Oslo, Norway; christopher.dirdal@sintef.no (C.A.D.); GeirUri.U.Jensen@sintef.no (G.U.J.); paul.thrane@sintef.no (P.C.V.T.); Hallvard.Angelskar@sintef.no (H.A.)

**Keywords:** metasurface fabrication, cryogenic etching, bosch process, deep reactive ion etching

## Abstract

The research field of metasurfaces has attracted considerable attention in recent years due to its high potential to achieve flat, ultrathin optical devices of high performance. Metasurfaces, consisting of artificial patterns of subwavelength dimensions, often require fabrication techniques with high aspect ratios (HARs). Bosch and Cryogenic methods are the best etching candidates of industrial relevance towards the fabrication of these nanostructures. In this paper, we present the fabrication of Silicon (Si) metalenses by the UV-Nanoimprint Lithography method and cryogenic Deep Reactive Ion Etching (DRIE) process and compare the results with the same structures manufactured by Bosch DRIE both in terms of technological achievements and lens efficiencies. The Cryo- and Bosch-etched lenses attain efficiencies of around 39% at wavelength λ = 1.50 µm and λ = 1.45 µm against a theoretical level of around 61% (for Si pillars on a Si substrate), respectively, and process modifications are suggested towards raising the efficiencies further. Our results indicate that some sidewall surface roughness of the Bosch DRIE is acceptable in metalense fabrication, as even significant sidewall surface roughness in a non-optimized Bosch process yields reasonable efficiency levels.

## 1. Introduction

The research field of metasurfaces represents a novel development within photonics where structured surfaces are used to manipulate light at an unprecedented level. Typically consisting of planar arrays of resonant structures which are small compared to the wavelength of the electromagnetic field, metasurfaces seem capable of almost arbitrary field control—the phase, polarization, amplitude and dispersion of the transmitted or reflected field interacting with the surface can almost be determined at will through clever design of the structure. Combined with the possibility of fabricating such structures using existing silicon processing techniques, these capabilities for unprecedented field control have made metasurfaces one of the most active research fields within photonics today.

In parallel with the drive towards demonstrating novel field control, the research field has recently intensified efforts towards bringing metasurface fabrication into the established silicon processing lines (e.g., of VECSEL lasers and optical detectors [1,2]). The goal of this direction is to offer a route towards dramatic miniaturization and integration of optical components (e.g., integrating flat lenses into optical detectors [2] or on of top diode lasers [1]). While optical systems have generally been big, heavy and expensive, metasurface fabrication in silicon fabs represents a new paradigm for optical systems whereby they can become small, lightweight and inexpensive. For this reason, metasurface research field was listed among the Top Ten Emerging Technologies 2019 by the World Economic Forum. It is currently generating significant industry interest with several emerging startups.

For high-performance dielectric metasurfaces, there is often a need for medium-to-high gap aspect ratios between dielectric pillars [3,4,5,6,7]. For instance, some dispersion engineering applications are limited by the achievable aspect ratios [5]. In the literature, some of the highest aspect ratios between metasurface dielectric pillars have been achieved by using a process relying on patterning of high aspect ratio resist holes and subsequent Atomic Layer Deposition (ALD) and lift-off [4,5,6]. While this technique achieves gaps as small as ~20 nm between pillars of heights in the order of 600 nm [4], its reliance on Electron Beam Lithography (EBL) for resist patterning and ALD for deposition of the pillars make it too slow for industrial metasurface fabrication. Two industrially relevant techniques which offer high aspect ratios are top-down etching processes by Bosch and Cryogenic Deep Reactive Ion Etching (DRIE). Bosch DRIE is readily available in most MEMS fabs and it relies on a three-step process to achieve high aspect ratio (HAR) structures—cyclical alternation between isotropic etching, passivation and de-passivation. The process leads to characteristic washboard-type surface roughness. In the literature it has sometimes been assumed that straight sidewalls are necessary [6] for high optical quality, which at first glance may seem to put the Bosch process at a disadvantage. However, we have recently argued and shown by simulations that the surface roughness can be compensated for, as long as the scallops are small relative to the wavelength [8]. Despite the availability of Bosch DRIE, the Cryogenic (Cryo) technique is also becoming more common in developing nanostructures for optical applications [9,10]. The cryogenic etching process is a single step continuous process using SF_6_ and O_2_ as process gases, performed at low temperatures, below −100 °C, which enable the formation and condensation of SiF_x_O_y_ on cooled surfaces, and act as a passivation layer. The O_2_ in the plasma is mainly responsible for the formation of the passivation layer by reacting with bare Si and SiF_4_ byproducts. By controlling the O_2_ concentration, the process can be adjusted to create vertical profiles, positive or negative slopes [11]. These advantages can be tracked back to the cryogenic process being continuous, with the passivation of the sidewalls taking place simultaneously, in contrast to the Bosch process, which requires separate passivation and etching steps. Sidewall profile control can be much more easily obtained just by adjusting O_2_ flow and the Inductively Coupled Plasma (ICP) generator power levels. Due to this fact, cryogenic etching processes can be employed for etching extremely small structures down to the resolution of electron beam lithography without undercutting the masking layer. In addition, the cryogenic SF_6_/O_2_ etching process is carbon-free and therefore extremely clean, without deposition of the polymer on the surfaces of the resulting nanopatterned structures and the chamber walls. However, Cryo etch has so far been less prevalent in industry than Bosch due to its dependence on very accurate temperature control of the wafer and etched structures towards achieving process controllability, uniformity and repeatability. There are nevertheless indications that Cryo is gaining interest also in industry [9]. Taking on the advantages of cryogenic etching, Yao and Wu demonstrated the feasibility of manufacturing an all-dielectric metasurface with positive sloped sidewalls, showing more than 90% reflection in the 600 to 800 nm wavelength range [12]. In this case, SiF_x_O_y_ passivation was not only used to control the slope angle, but also to protect the softer materials like SiO_2_ and Si_3_N_4_ deposited on the bottom a-Si layer.

Removing the scallops which form during the Bosch process can be beneficial when one has to achieve extremely narrow gaps or if the pillar dimensions are small compared to the scallop depths. Cryogenic silicon etching enabled Li et al. to successfully fabricate different types of metasurfaces ranging from nanopillars to hollow cylinders and honeycomb networks with extreme dimensions, standing up to 200 nm in height and having a sidewall thickness of only 10 nm [9]. This approach allows exploration of new types of nano-scale features for manufacturing metasurfaces.

In this publication we present metastructures fabricated by cryogenic DRIE and compare them with earlier fabricated metastructures using Bosch DRIE [8]. The resulting optical performance of the metasurfaces for the smooth (cryo) and washboard type (Bosch) sidewalls resulting from each technique are considered. As Bosch and increasingly Cryogenic process are etching techniques of industrial relevance towards the fabrication of HAR metasurfaces, we have chosen to also use the high-throughput UV-Nanoimprint lithography technique to pattern the wafers. Until recently, virtually all high-end HAR metasurfaces have been fabricated using Electron Beam Lithography (EBL) patterning, which is comparatively slow and challenging to use for cost-effective large area metasurface fabrication.

## 2. Materials and Methods

### 2.1. Optical Design and Simulations

**Figure 1:**
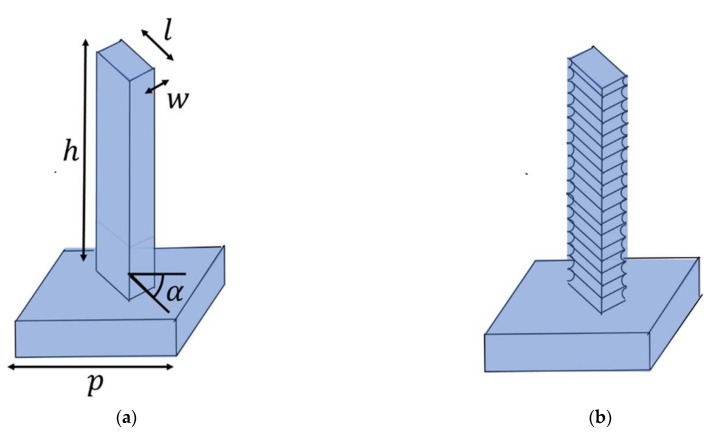
Sketches of metasurface unit cells arising from (**a**) Cryo DRIE where the sidewalls are smooth and (**b**) Bosch DRIE where the sidewalls have a characteristic washboard pattern in which scallops arise from cyclically etching isotropically, passivating and de-passivating.

The surface structure of the metasurface consists of rectangular pillars in silicon which act as pointwise phase shifters (Figure 1a). While early metasurfaces typically utilized plasmonic structures, dielectric structures have become popular owing to less issues with losses. The phase $\phi \left(r\right)$ imposed by each rectangular pillar at coordinate r is chosen to sample the hyperbolic phase function of a lens,

$$\phi \left(r\right)=-\frac{2\pi }{\lambda }\left(\sqrt{r^{2}+f^{2}}-f\right),$$

for the target wavelength λ=1.55 µm where normally the incident field from below is focused to a point a distance f=10 mm above the metasurface. In other words, the metasurface acts as a lens, or metalens. The ability of the structure to shift the phase between 0 and 2π is achieved based on the geometric phase principle [4,6,13,14] where the metastructure (i) converts between circular polarization states (acting as a half-wave plate), and (ii) applies a phase shift to the converted circular polarization state which is equal to twice the angle of rotation of each rectangle. Hence, the unit cell structures can be identical in geometry apart from a rotation angle—which is beneficial towards the optimization of the UV-NIL process as discussed in Section 2.2. For an identical array of pillars, a simple expression for the transmitted field can be derived in terms of a superposition of circular polarization states

$$|E_{\mathrm{out}}\rangle =\frac{t_{x}+t_{y}}{2}|R\rangle +\frac{t_{x}-t_{y}}{2}\exp\left(-i2\alpha \right)|L\rangle ,$$

where the incoming field is assumed to be right-hand circular polarized |R〉, and cross-polarized field is designated |L〉. The complex transmission coefficients of the metasurface array are designated $t_{x}$ and $t_{y}$ for linearly polarized light along orthogonal directions $\hat{x}$ and $\hat{y}$, perpendicular to the optical axis, which is normally incident on the metasurface. It is observed that the cross-polarized term attains a phase of 2α, where α is the rotation angle of the pillar (as shown in Figure 1a). A good structural design aims to ensure that $t_{y}=-t_{x}\equiv t,$ thereby making the transmitted field fully cross-polarized. When this is achieved, the output field becomes

$$|E_{\mathrm{out}}\rangle =t\exp\left(-i2\alpha \right)|L\rangle$$

for which the transmitted efficiency can be estimated to be in the order of ${\left|t\right|}^{2}\approx 69\%$ in the case of silicon pillars on a silicon substrate. This estimate disregards, e.g., diffraction scattering due to the structure, and is simply based on the fact that a bare Silicon substrate experiences around 31% reflectivity at the Si-air interface. Following the same reasoning, the efficiency could be raised to above 95% if the Si pillars instead were fabricated on a quartz wafer. To achieve maximum transmitted efficiency for the target wavelength of λ=1.55 µm, the rectangle width, length, height, and periodicity have been found to be w=230 nm, l=230 nm, h=1200 nm, and p=835 nm, respectively, based on sweep simulations (Finite Difference Time Domain and Rigorously Coupled Wave Analysis methods). It is worth noting that deviations from these target dimensions during fabrication primarily influence the transmission coefficients $t_{x}$ and $t_{y}$, and not directly the added phase 2α. As a result, the errors in fabrication may lower the efficiency of the lens (primarily through a reduction in cross-polarization efficiency), but would not necessarily prevent diffraction limited focusing. This is a clear advantage of utilizing the geometric phase principle, yielding structures that are quite robust towards fabrication imperfections.

While cryogenic etching can create the smooth sidewalls of the designed pillars, the cyclical isotropic etch, passivation and de-passivation steps of the Bosch process will inevitably introduce “washboard”-type sidewall surface roughness to the pillars (Figure 1b). The “washboard”-type patterns are characterized by a scallop depth, typically in the order of 15–80 nm in the fabrication presented here. The scallops reduce the pillars’ volume. For significantly subwavelength scallops such as these, they can be understood as thereby reducing the effective optical thickness of the pillars. Hence, in order to roughly compensate for the scallops, the simplest strategy is to increase the lateral dimensions corresponding to the volume loss. This assumes that the gap between the pillars is sufficient for such an expansion—thereby providing a limit for the achievable gap aspect ratio achievable for a given scallop depth. For the Cryo process, no such considerations need to be made, as there are no scallops to compensate for.

Simulations have been conducted using Rigorously Coupled Wave Analysis (RCWA) in the GD-Calc implementation and Finite Difference Time Domain (FDTD) in the optiFDTD implementation. The RCWA and FDTD simulations have been verified against each other for the smooth-walled pillars. The OptiFDTD software (version 13.0.2.892 by Optiwave, Ottawa, ON, Canada) has been useful in drawing more complex structures (i.e., the Bosch washboard scallops). More details on the operational principle of the lenses and the simulation work can be found in our previous work [8].

### 2.2. Resist Mask Fabrication Using UV-Nanolithography

The resist mask was made by first priming the wafer with mr-APS1 by Micro Resist Technology Berlin, Germany, then spinning with Micro Resist Technology mr-NIL210-200 nm at 3000 RPM for 60 s, then finally roll-on imprinting using a PFPE stamp (Fomblin MD-40 by Solvay, Brussels, Belgium) on a plastic carrier foil and curing by UV-exposure was conducted on an EVG620 Smart NIL system by EVG, St. Florian am Inn, Austria. The stamp was fabricated from an Si wafer with rectangular pillars protruding from the surface of nominal height 500 nm. The unit cells within each metalens chip have identical filling factors, since the pattern consists of identical rectangles which are rotated differently. This simplifies the task of achieving uniformity in the residual layer thickness between resist pillars.

Different stamps were used for the Bosch and Cryo processed wafers, as these experiments were separated in time. During the UV-NIL processing of the latter, some form of bubbles appeared in the resist. The cause of these bubbles is not fully understood, but it could possibly be due to aging of the stamp material. More details on the development of the UV-NIL process can be found in our previous work [8].

### 2.3. Bosch Deep Reactive Ion Etching

**Figure 2:**
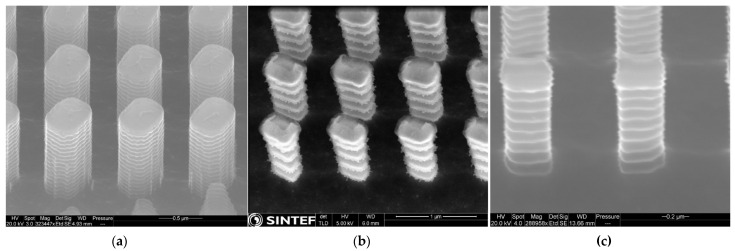
SEM images of the Bosch fabricated structures—(**a**) structures arising from the Bosch 1 process steps, (**b**) structures arising from the Bosch 2 process steps, and (**c**) structures arising from the Bosch 3 process steps. Note that in (**b**) the pillars still have a resist cap and possible polymer on the sidewalls.

**Figure 3:**
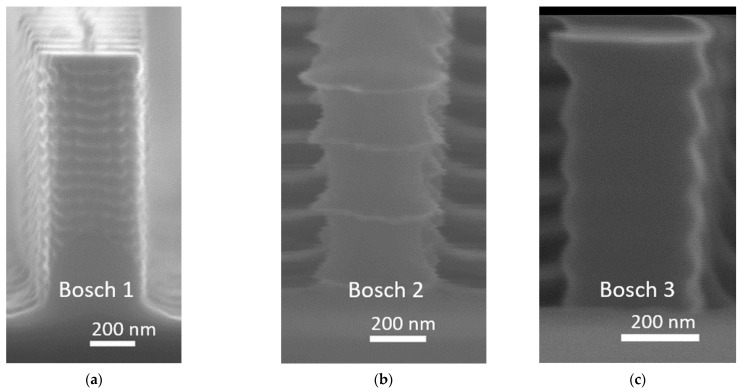
Bosch fabricated pillars. After removal of the residual UV-NIL resist layer, Bosch etching was conducted with the following parameters—(**a**) SF_6_ pulses of time 3.0 s and SF_6_ process pressure of 35 mTorr; (**b**) SF_6_ pulse of time 4.0 s and SF_6_ pressure of 60 mTorr; (**c**) SF_6_ pulse of time 3.0 s and SF_6_ pressure of 60 mTorr.

The dry etching was performed on a Rapier Si DRIE process module by SPTS, Newport, UK. The residual layer of the imprinted resist was first removed by a continuous, directional etch with Ar/O_2_/C_4_F_8_ plasma at a platen power of 450 W. It turned out that the resist pillars have a top-hat shape, in which rounding occurs at the base of each pillar [8], which is also commonly observed literature [10,15,16]. As discussed in [8], we believe that the resulting resist “lip” surrounding the base likely originates from equivalent rounding at the base of the Si pillars in the master wafer, although it is difficult to observe this in SEM without performing destructive cross-sectional analysis of the master wafer. The consequence of this rounding effect for the Bosch processing conducted is to increase the effective dimensions of the mask. A substantial overetch was therefore used to remove as much as possible of the resist lip that surrounds each pillar’s bottom. After residual layer removal, a pulsed Bosch Deep Reactive Ion Etch (DRIE) was conducted (with repeating cycles of polymer deposition, de-passivation, and SF6 silicon etch). The first Bosch process (Bosch 1) was conducted with SF6 pulses of time 3.0 s and SF6 process pressure of 35 mTorr. The resulting structure is shown in Figure 2a and Figure 3a. The lateral dimensions were found to be 420 × 530 nm (measured between the tops of the washboard pattern) with scallop depths of around 14 nm (i.e., the lateral distance into the Si pillars from the peak to bottom of the washboard pattern). The resulting dimensions overshoot the target dimensions by approximately 190 and 176 nm in the width and length directions, making these metasurface structures unsuited for operating with wavelengths around the target wavelength of 1.55 µm. This overshoot is likely explained by the initial overetch not removing enough of the resist lip. To circumvent the issue without fabricating a new master-wafer, we attempted two more runs in which the scallop depths were ramped up considerably—the idea being to reduce the effective optical dimensions of the Si pillars. In the first of these attempts (Bosch 2) the scallop sizes were increased to 76.5 nm by increasing the SF6 pulse time to 4.0 s and SF6 pressure to 60 mTorr. This gave lateral dimensions of around 307 × 460 nm, as measured from peak-to-peak between the washboard patterns. The resulting structure is shown in Figure 2b and Figure 3b. In the second attempt, we instead used slightly smaller scallop depths of 44 nm by reducing again the SF6 pulse time to 3.0 s and keeping the SF6 pressure at 60 mTorr. Thereafter, a thermal oxidation step and oxide strip was conducted to reduce the dimensions to 210 × 320 nm and relative scallop depths to 29 nm. The resulting structures are shown in Figure 2c and Figure 3c.

### 2.4. Cryogenic Deep Reactive Ion Etching

**Figure 4:**
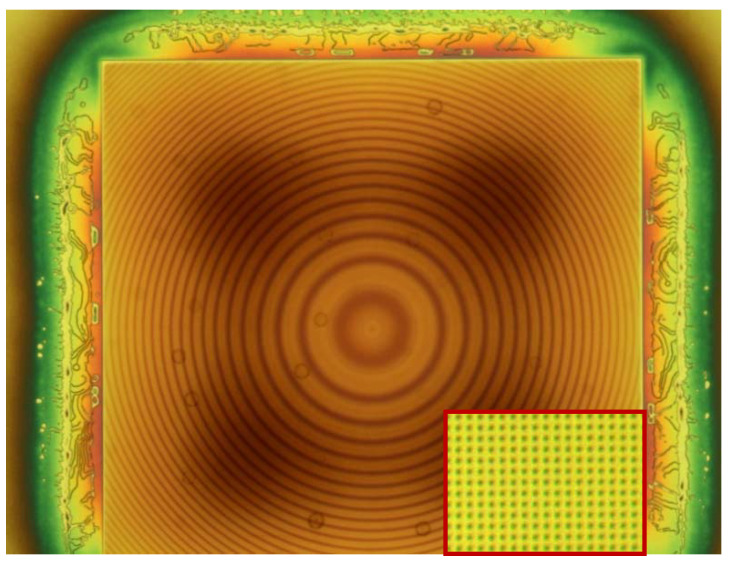
Optical microscopy image of the metalens after 30 s. of oxygen plasma (during the RL removing process).

In order to transfer the pattern from the mr-NIL210-200 nm resist to the silicon wafer, it was first required to remove the residual layer (RL—the resist formed between imprinted pillars). This process was performed by oxygen plasma, using an RIE system (Etchlab SI 220-Sentech Instruments, Berlin, Germany). The values of this recipe’s parameters included a pressure of 150 mTorr, an ICP power setting of 200 W and 50 sccm O_2_ flow. Due to the significant non-uniformity thickness of the residual layer between wafers, oxygen etching was performed in subsequent steps with optical investigations in between (Figure 4), which allowed us to evaluate the total required etching time.

**Figure 5:**
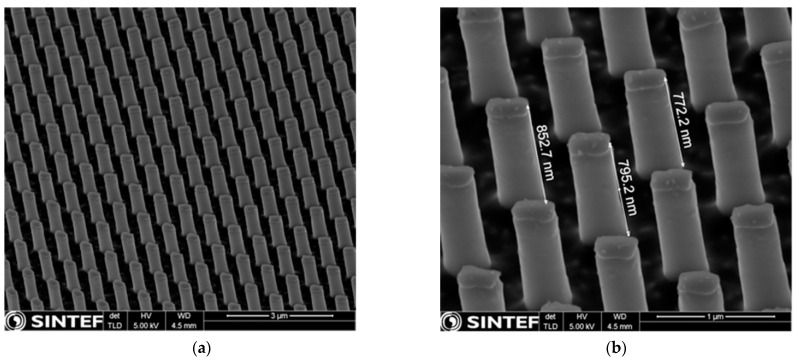
Cross-sectional view of SEM images of the nanopatterned silicon after cryogenic etching process (tilt 45°)—(**a**) the fidelity of the nanopillars (**b**) with corresponding measured heights.

For most samples, we allowed for two steps of 15 s each for the complete removal of the residual layer, though in some cases additional plasma exposure was required. The subsequent etching of the silicon wafers was achieved by using the cryogenic process, performed in an Inductively Coupled Plasma Reactive Ion Etching (ICP-RIE) system—PlasmaLab 100 (Oxford Instruments Ltd, Yatton, UK). The process was carried out at −115 °C, using the ICP power of 1200 W, RF power of 3 W, a pressure of 7.5 mTorr and 60 sccm SF_6_ flow and 8 sccm O_2_ flow. Figure 5a shows that high pattern fidelity of the silicon pillars was achieved. Vertical sidewalls and heights of around 800 nm were observed Figure 5b. At the end of the technological flow, the mr-NIL210-200 nm resist was completely removed in oxygen plasma (5 min process).

## 3. Results and Discussions

Table 1 lists and compares the lateral dimensions of the different metastructures achieved from the fabrication steps discussed above. For the Bosch processed structures, the lateral dimensions are measured from peak-to-peak on the washboard surface patterns. The cryo-processed structures match the target lateral dimensions of the metastructure well, being roughly 235 × 355 nm, however being slightly short of the desired height with around 800 nm.

For the Cryo-processed structures, the masking pattern shrunk in size by ~20% after O_2_ plasma ashing. This shrinkage, along with rounding of the corners of the patterned structures, was caused by using a pure O_2_ for the plasma ashing process, which is slightly isotropic, etching away not only the residual layer but the sidewalls of the masking patterns as well. These new slightly reduced dimensions were subsequent transferred to the silicon layer in the cryogenic etching process.

**Figure 6:**
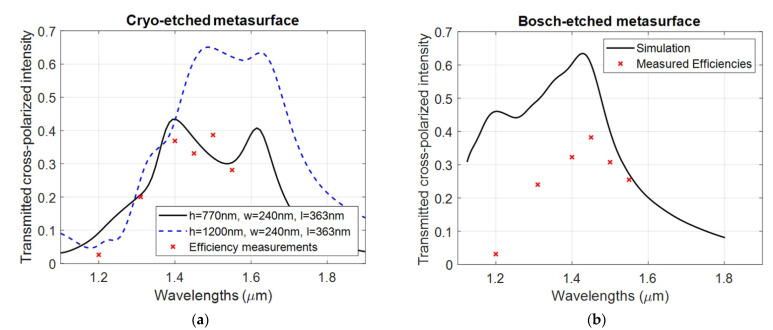
Plots of simulation curves based on dimensions found from SEM images and datapoints from efficiency measurements. (**a**) Results for cryo-etched metasurface, using the Rigorously Coupled Wave Analysis (RCWA) simulation method for a pillar of dimensions h=770 nm, w=240 nm, l=363 nm and p=835 nm. (**b**) Results of the second Bosch run for which the Bosch scallops were dramatically increased.

Regarding the obtained height of the silicon nanopillars, the performed cryogenic etching recipe should have provided a depth of 1200 nm. However, the obtained depth of only 800 nm was probably caused by the incomplete removal of the residual layer, resulting in a lower efficiency than expected. This issue can be overcome either by accurately measuring the residual layer or by performing a longer cryogenic etching process. Figure 6a compares the optical efficiency of the Cryo-etched metalens with the corresponding simulations for two heights. The transmission curve (solid curve) fits qualitatively with the measured efficiency of the cryo-etched metasurface. The simulation curves indicate that the efficiency can be raised to the design efficiency by raising the heights to the target height of 1200 nm through a longer etch step. Hence, the process parameters for the UV-NIL and Cryo etch process have been close to achieving the target structures in this case.

It should be pointed out that during the RL removing process, we observed considerable differences of the residual layer thickness between wafers. A simple solution for improving process control could be the automated dispensing of resist in the UV-NIL process.

The optical efficiency measurements were done as described in [8], except that the light source was a fiber-coupled thermal lamp (SLS 201 by Thorlabs, Newton, NJ, USA). After emerging from the multi-mode fiber, the light was collimated using an NIR antireflection-coated aspherical lens, before passing through an aperture with diameter 0.9 mm. After the aperture, the beam passed through the backside of the Si-substrate before being focused by the metalens. The focal point was imaged using a ×20 infinity corrected NIR microscope objective (Mitutoyo Plan Apo by Mitutoyo, Kanagawa, Japan), tube lens and NIR camera (NiT WiDy SenS 640 V-ST by New Imaging Technologies, Verrières le Buisson, France). To measure the efficiencies at several wavelengths between 1.2 and 1.55 um, narrowband filters with a full width half maximum of 12 nm were used to filter the thermal light prior to the light being coupled into the multi-mode fiber. To be able to compare efficiencies at different wavelengths, the output of the filtered thermal source was measured using a power meter for each of the wavelengths, and the camera efficiency was subsequently determined by imaging the source fiber tip directly for each wavelength. The absolute efficiency value was acquired by using results from [8], where metalenses fabricated using the Bosch process were measured to have an efficiency of 26% at 1.55 um by comparing to an anti-reflection-coated aspherical lens.

**Figure 7:**
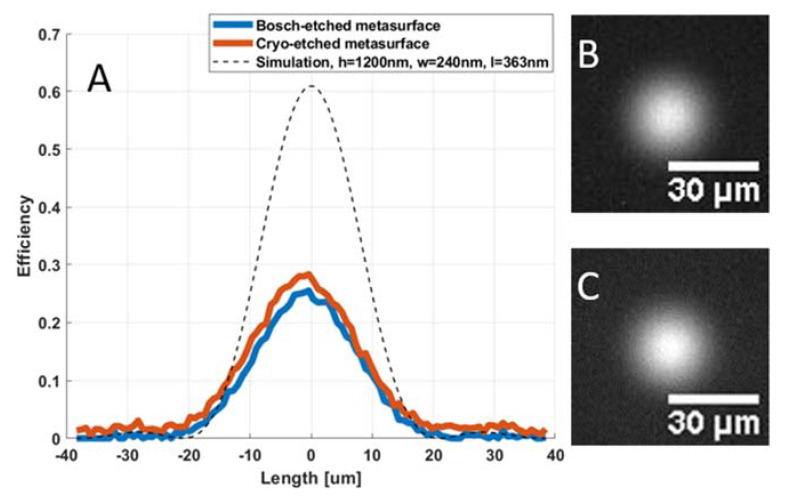
(**A**) Measured focal spot profiles of a cryo- and a Bosch-etched metalens (blue and orange respectively). The dashed line shows the theoretical focal spot of a diffraction-limited lens with efficiency equal to the cross-polarization efficiency expected from the simulated design structures. To the right are images of the focal spot for a Bosch-etched (**B**) and cryo-etched (**C**) metalens. The focal length of all the lenses was 10 mm and an aperture with diameter 0.9 mm was placed in front of the lenses. The incident light in both the focal spot profiles and images is thermal light filtered using a 12 nm full width at half maximum (FWHM) bandpass filer centered at 1550 nm, and the resulting low intensities are the cause of the noise that can be observed in the measurements.

The measured focal spots of the metalenses are shown in Figure 7. As expected, the use of the geometric phase principle leads to close-to-diffraction limited focusing despite certain deviations in the fabricated structures from the target. High aspect rectangular dielectric pillar metalenses of the kind we have fabricated are known to typically exhibit strong chromatic aberrations [5] and the hyperbolic lens function utilized is known to lead to significant coma [17], however the current metalenses have not been thoroughly characterized for aberrations. The metalenses were designed to have a focal length of 10 mm for *λ* = 1.55 µm, but the actual focal length is wavelength-dependent, increasing to around 12 mm as the wavelength is reduced to *λ* = 1.31 µm, as expected from the lens phase function, which predicts that the focal length scales inversely with the wavelength. The uncertainty of the efficiency measurements is largely determined by the absolute efficiency comparison with the aspherical lens, but is also affected by the wavelength dependency measurements described above. Based on the repeatability of our measurements, we estimate the accuracy of the results to be in the order of 5%.

The Rigorously Coupled Wave Analysis (RCWA) method was used in the GD-Calc implementation to simulate a unit cell consisting of an individual pillar and 10 diffraction orders that were used.

Due to the aforementioned resist broadening at the base of the UV-NIL-imprinted resist pillars, the intended Bosch process (Bosch 1) ended up with dimensions significantly larger than the target dimensions. As a result, it has a poor optical response within the wavelength bandwidth of interest $\lambda \in \left(1.31\;µm,\;1.55\;µm\right)$. Nonetheless, had the lateral dimensions been scaled correctly (by fine-tuning the resist opening step, or reducing the lateral dimensions in the master) simulations indicate that the structure would have given a high-quality optical performance. It is worth noting that the resist broadening likely can be dealt with by using a suitable residual layer removal process, as seems to have been achieved in the case of the cryogenic metasurface fabrication. Also, the authors of [15] present an optimized process which removes such resist broadening. Another possible route towards avoiding resist broadening may be the fabrications of an Si-master consisting of holes instead of protrusions (and thereby fabricate a stamp from, e.g., resist imprinted by the master) whereby the rounding effect may then occur at the top of the pillars rather than at the base.

**Figure 8:**
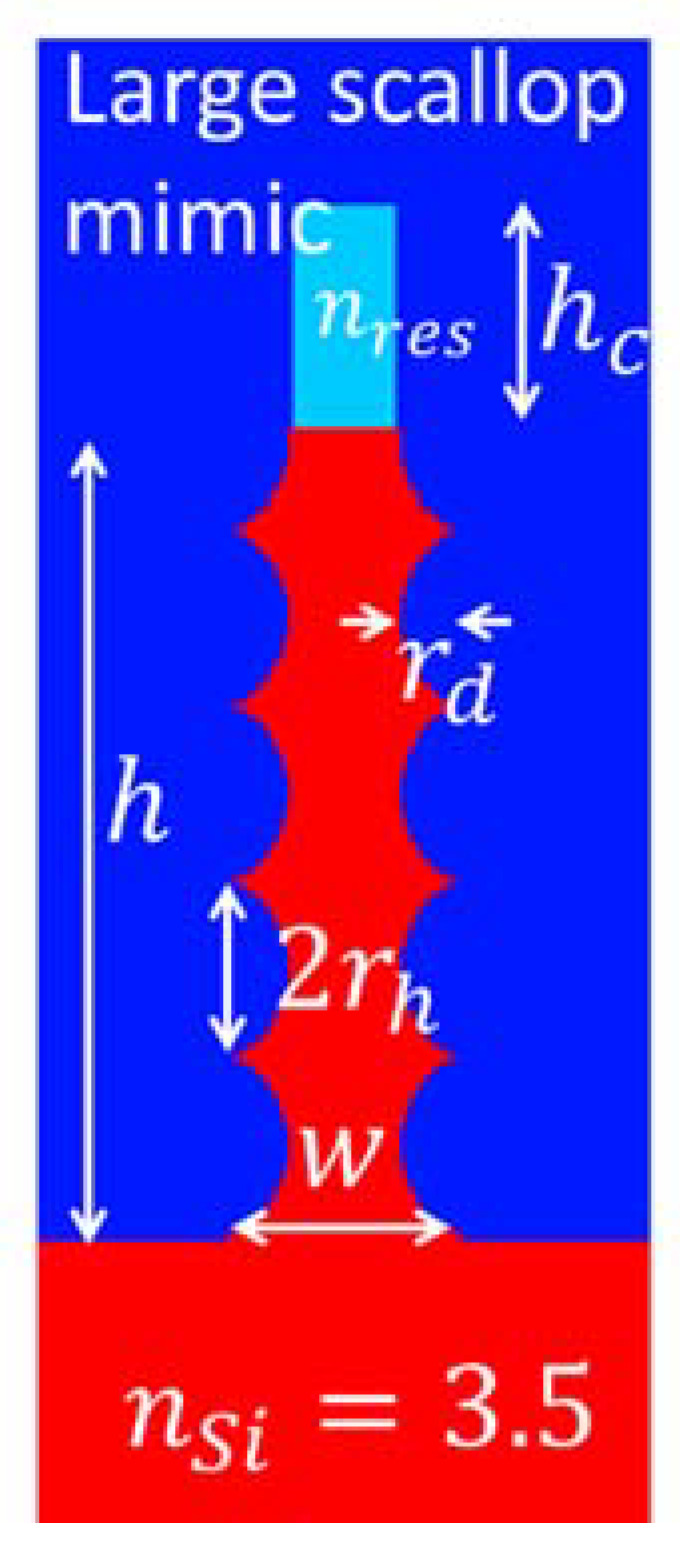
Simulation structure mimicking the features of the Bosch lens—large oval scallops and a resist cap that remained after post-dicing piranha treatment.

In this paper, the strategies employed to reduce the lateral dimensions in Bosch 2 and Bosch 3 gave dimensions closer to the target, although also for Bosch 3 the resulting dimensions place the peak optical performance outside the wavelength bandwidth of interest. Although the peak-to-peak values of the washboard patterns (Figure 3c) are only slightly smaller than the target value, the effective optical dimensions are smaller due to the pillar volume lost to the scallops in between. The measured lens efficiencies of resulting dimensions of Bosch 2 are shown in Figure 6b. A simulation curve is added for a rectangular structure with elliptical washboard surface patterns intended to mimic the structures as seen in SEM images. The simulation geometry is shown in Figure 8, for which the parameter values are w=307nm, l=406nm, $r_{h}=119\mathrm{nm}$, $r_{d}=76.5\mathrm{nm}$, h=1100nm, $h_{c}=300\mathrm{nm}$ and $n_{res}=1.44$, i.e., a pillar with rectangular cross section of varying size along the pillar. A resist cap is included due to the fact that the attempted post-dicing piranha removal of the resist was unsuccessful, but simulations with and without this cap indicate that it has little influence on the optical performance. The Finite Difference Time Domain method (FDTD) in the OptiFDTD implementation was used for the relative ease at which complex geometries can be drawn. A single pillar was simulated with periodic boundary conditions laterally surrounding the unit cell, and perfectly matched layers above and below. A source pulse width FWHM of $6.23\cdot 10^{-15}\;s$ for the target wavelength of λ=1.41 µm was used and a simulation time of 25,000 timesteps, each being $\Delta t=5.5\cdot 10^{-18}s$ long. A non-uniform mesh grid was used to allow for a fine mesh nearby to the Si pillars (to properly resolve the scallops). For the directions orthogonal to the pillar heights (i.e., orthogonal to the optical axis) the FDTD mesh grid interspacing varied between 3 nm≤Δx,Δy≤6 nm, while along the optical axis the mesh grid interspacing varied between 3 nm≤Δz≤10 nm.

While the wavelength for peak optical efficiency coincides well with the measurements, the measured efficiency is somewhat lower than predicted. The geometry of the pillar is, however, not easily simulated accurately. Also, these pillars were not properly stripped for resist and etch polymer prior to optical testing, although this is at least partially accounted for in the simulation. Despite there potentially being several sources of error with the simulations, a part of the discrepancy is possibly explained by rounding effects within the groves of the washboard patterns, where it seems possible that the pillar cross-section ceases to be rectangular and rather begins to become more circular (see Figure 3b). Circular cross-sections are incapable of converting between circular polarization states, hence reducing the efficiency of the metasurface. A reduction in the cross-polarization efficiency may, for instance, be observed by simulating a pillar consisting of a stack of alternating circular disks and rectangular bricks—an extreme version of what we see in Figure 3b which has a simpler shape, which is easy to draw for simulation purposes. Despite the drawbacks of the resulting structures in the Bosch 2 fabrication, it is worth noting that the optical performance is comparable to that achieved by the Cryo process (Figure 6a), indicating that even scallops as large as those seen in Figure 3b allow for a metalens with reasonable efficiency. Indeed, the simulation curve indicates that a scallop pillar with rectangular cross-section should be able to achieve more than 60% efficiency, i.e., that the scallops do not present a problem in themselves.

These metalenses have been made to operate for NIR, while many optical imaging applications exist for metalenses in VIS. Although the use of Si metastructures is often avoided in VIS due to absorption, absorption can be significantly reduced by switching to a quartz substrate. Transitioning from NIR (1.55 µm) to VIS (~500 nm) will, however, generally involve reducing the structural dimensions (roughly by a factor of three) for which fabrication is potentially more challenging and in need of further process development.

## 4. Conclusions

We demonstrated the fabrication of metalenses using a combination of UV-NIL and Cryogenic-DRIE processes, and compared the results to metalenses fabricated by UV-NIL and Bosch DRIE. UV-Nanoimprint lithography was chosen as a patterning technique for its potential to achieve large-area nanopatterning at affordable operating costs. Hence, this publication has aimed to develop processes that are relevant for industrial metasurface fabrication. The Cryo-DRIE process enabled the fabrication of silicon nanopillars with smooth vertical profiles. Optical measurements of the Cryo metalenses were performed at discrete wavelengths in the interval λ ∈ (1.31 µm, 1.55 µm), achieving a peak efficiency of around 39%. Simulation results indicated that the efficiency can be raised to the target efficiency of around 65% by increasing the height of the silicon nanopillars from 800 to 1200 nm.

The efficiency of the Bosch-fabricated metalenses was found to be similar to that of the Cryo-etched lenses. The Bosch-fabricated metalenses ended up with dimensions larger than the target values due to resist broadening occurring at the base of the UV-NIL-imprinted resist nanopillars. To circumvent the issue, the scallop sizes were increased dramatically to reduce the effective optical dimensions of the Bosch pillars, i.e., although the peak-to-peak values of the resulting washboard patterns are larger than the target value, the effective optical dimensions are smaller due to the pillar volume lost to the scallops in between. The measured efficiency of the Bosch metalenses was lower than simulated. While there are several possible sources of error for the simulated curves in the case of the Bosch-processed structure, a part of the explanation may be pillar rounding within the groves of the washboard patterns. Indeed, simulations indicate that scallops in themselves are not a problem, and that a pillar with large scallops and rectangular cross-sections should be able to achieve more than 60% efficiency (i.e., the theoretical efficiency for a smooth structure with an Si substrate).

Process optimization strategies were discussed for both deep reactive ion etching techniques to improve metalens efficiency. Importantly, the resist broadening effect, occurring during the nanoimprint process, could potentially have been avoided by using a residual layer removing process similar to that used in the case of the cryogenic metasurface fabrication. Other possible routes could be to account for the broadening in the design of the master, or to avoid it by fabricating the Si-master consisting of holes instead of pillars.

## Figures and Tables

**Table 1:** Dimensions of nominal patterns in master and fabricated structures.

DRIE Method	Scallop Sizes (nm)	Nominal Dimensions on Master (nm)		Metastructure Dimensions (nm)		Discrepancy (%)	
		Width	Length	Width	Length	Width	Length
Cry	NA	351	475	235	355	−33	−25
Bosch 1	14	237	361	420	530	77	47
Bosch 2	76.5	237	361	307	460	30	27
Bosch 3	44/29	237	361	210	320	−11	−11

## Data Availability

Not applicable.
